# A minute fraction of α-synuclein in extracellular vesicles may be a major contributor to α-synuclein spreading following autophagy inhibition

**DOI:** 10.3389/fnmol.2022.1001382

**Published:** 2022-09-28

**Authors:** Hash Brown Taha, Brian Kearney, Gal Bitan

**Affiliations:** ^1^Department of Integrative Biology and Physiology, University of California, Los Angeles, Los Angeles, CA, United States; ^2^Department of Neurology, David Geffen School of Medicine, University of California, Los Angeles, Los Angeles, CA, United States; ^3^Brain Research Institute, University of California, Los Angeles, Los Angeles, CA, United States; ^4^Molecular Biology Institute, University of California, Los Angeles, Los Angeles, CA, United States

**Keywords:** extracellular vesicle (EV), autophagy, synucleinopathy, alpha-synclein, p62, post translational modification (PTM)

Reduced protein clearance, particularly of the autophagy-lysosome pathway (ALP), leads to increased release of aggregated α-synuclein in synucleinopathies. A recent paper (Oh et al., 2022) has suggested a new mechanism that may contribute to these processes. We discuss here these new findings and their implications for our understanding of the mechanisms of pathology spread in the brain of patients with synucleinopathies.

With advances in medical care and technology resulting in the extension of lifespan, comes an increased risk for neurodegenerative diseases because age is a predominant risk factor for development of many of these disorders. By 2030, 1 in 5 individuals is predicted to be over the age of 65, increasing the projected incidence and prevalence of neurodegenerative diseases (Hou et al., 2019). Synucleinopathies, such as Parkinson's disease (PD) and Lewy body dementia (LBD), are a group of neurodegenerative diseases marked by extensive, abnormal accumulation of aggregated intracellular α-synuclein in neurons. In PD and LBD, α-synuclein aggregates form Lewy bodies and Lewy neurites, which are pathological hallmark of these diseases. Though α-synuclein is the main component of Lewy bodies, they also contain other proteins. One of these proteins is p62, a classical receptor of autophagy and a multifunctional protein located throughout the cell and involved in many signal transduction pathways (Zatloukal et al., 2002).

Eukaryotic cells rely on two primary mechanisms for degradation and recycling of proteins: the ubiquitin-proteasome system (UPS) and the ALP (Ciechanover and Kwon, 2015). Though both pathways help maintain proteostasis, only the ALP participates in the clearance of insoluble protein aggregates. In PD and LBD, failure of the ALP is a key component of the mechanisms leading to accumulation of Lewy bodies and neurodegeneration (Kocaturk and Gozuacik, 2018). Under these conditions, distressed cells attempt to remove the excess α-synuclein using several mechanisms (Hijaz and Volpicelli-Daley, 2020; Bras and Outeiro, 2021), including secretion into the extracellular space through exocytosis (Lee, 2005; Emmanouilidou and Vekrellis, 2016), secretion in extracellular vesicles (EVs), such as exosomes (Emmanouilidou et al., 2010; Alvarez-Erviti et al., 2011; Danzer et al., 2012), and possibly directly to neighboring cells through nanotubes (Abounit et al., 2016; Dieriks et al., 2017), all of which contribute to the spread of pathological α-synuclein in the brain. Extracellular forms of α-synucleins propagate between various types of cells, bind to cell surface receptors and transmit signals, regulating numerous intracellular processes (Surguchev et al., 2019). The increased transfer of α-synuclein in EVs in particular may be a double-edged sword: on one hand, ridding the affected cells of misfolded, dangerous proteins, yet on the other, increasing the risk of transferring pathologic protein seeds to neighboring cells, leading to neurodegeneration (Vargas et al., 2019).

It is hypothesized that various mechanisms may trigger and/or contribute to ALP dysfunction, including an increase in reactive oxygen and nitrogen species (ROS/RNS) (Sarkar et al., 2011) and reduction of glucocerebrosidase enzymatic function (Mazzulli et al., 2011), leading to α-synuclein accumulation and secretion. However, the precise molecular mechanisms linking oxidative stress due to increased levels of ROS/RNS to ALP dysfunction and α-synuclein accumulation and secretion have yet to be elucidated.

Recently, Oh et al. (2022) addressed this knowledge gap by testing the effect of S-nitrosylated p62 (SNO-p62) on autophagic flux and subsequent α-synuclein secretion in cell-culture and mouse models (Figure 1). This line of investigation was pursued following their realization that p62 contains a motif that makes it susceptible to S-nitrosylation. p62 is an important regulator of autophagic flux, which among its many roles in the cell, helps maintain α-synuclein homeostasis (Tanji et al., 2015). Thus, Oh et al. tackled several questions, including whether SNO-p62: (1) is found in the brain of patients with synucleinopathies and synucleinopathy disease models; (2) modulates autophagic flux, and (3) affects secretion and cell-to-cell spread of α-synuclein in EV-dependent and EV-independent pathways.

**Figure 1:**
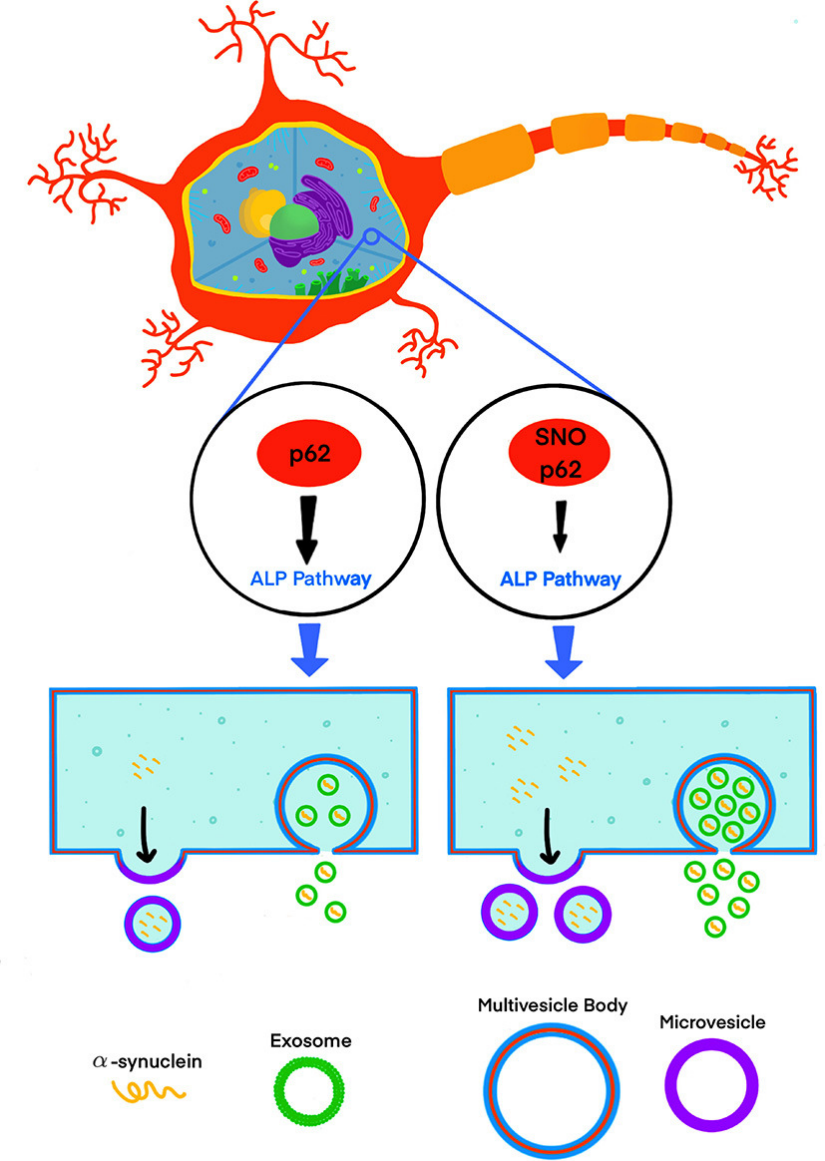
S-nitrosylation of p62 (SNO-p62) attenuates the autophagy-lysosomal-pathway (ALP), increasing release of α-synuclein in EVs.

Oh et al. demonstrated first that both endogenous nitric oxide (NO) and exogenous S-nitrosocysteine (SNOC) S-nitrosylated p62 in SH-SY5Y cells and human induced pluripotent stem-cell (hiPSC)-derived neurons. Importantly, they also showed that SNO-p62 was increased in post-mortem PD and LBD brains compared to non-diseased brains, suggesting that this post-translational modification is clinically relevant in synucleinopathies. They then found SNO-p62 to be increased in models of α-synucleinopathy, including Thy1-promoter-driven α-syn-overexpressing mice (Rockenstein et al., 2002; Chesselet et al., 2012) and hiPSCs expressing the familial PD-linked A53T variant of α-synuclein, compared to matched controls.

LC3 is a key protein orchestrating autophagosome biogenesis, which interacts with multiple proteins, including p62, via their LC3-interacting region (LIR) (Pankiv et al., 2007; Kraft et al., 2010). Using SH-SY5Y cells and co-immunoprecipitation, Oh et al. demonstrated that treatment with SNOC increased interactions between p62 and LC3, suggesting that S-nitrosylation was responsible for the increased affinity. They asked then what the specific site of S-nitrosylation might be. The LIR of p62 contains a single cysteine residue, Cys331, which the authors suspected to be the primary site of S-nitrosylation. To test this hypothesis, they created a C-terminal fragment of p62, spanning residues 230–440, containing an intact LIR motif. Upon substitution of Cys331 by Ala in this fragment, co-immunoprecipitation data of the tagged p62 construct to LC3 revealed a 75% reduction in S-nitrosylated p62 (230–440) after exposure to SNOC, suggesting that Cys331 indeed was the primary site for S-nitrosylation.

At this point, the investigation took an unexpected turn. The findings described above motivated the authors to use a full-length p62 (C331A) model in subsequent experiments, presumably with the expectation that removal of the Cys side chain, and hence the ability to nitrosylate this side chain, would abolish the increased binding of SNO-p62 to LC3. Surprisingly, though the methyl sidechain of Ala is distinct in its size, electronic character, and charge distribution from the nitrosylated methylenesulfhydryl sidechain of NO-Cys, rather than eliminating the increased affinity of p62 for LC3, p62 (C331A) phenotypically copied the increased interaction between SNO-p62 and LC3 and the subsequent inhibition of autophagic flux. Although the mechanism underlying this observed phenotypical similarity is not clear, this finding suggested that p62 (C331A) could be used in place of SNO-p62, eliminating the need to S-nitrosylate p62, which might generate confounding effects due to nitrosylation of other targets. We believe that further studies are needed to explore how the removal of the S-NO group achieves the same end result—increasing the strength of the interaction between the p62 LIR and LC3, despite the substantial structural and electronic difference between Ala and NO-Cys.

Interestingly, Oh et al. showed that although 99.99% of the secreted α-synuclein in the media of p62 (C331A)-expressing SH-SY5Y cells was not associated with EVs, secretion of EVs containing α-synuclein increased 3.3-fold in comparison to a 1.6-fold increase in EV-independent pathways, suggesting that if increased cell-to-cell transfer is observed, a minute fraction, 0.01% of EV-associated α-synuclein, might be an important contributor to this increase. To assess cell-to-cell spread, Oh et al. used a dual-cell bimolecular fluorescence complementation assay. This system uses structural complementation of two Venus protein fragments conjugated to α-synuclein and expressed separately, so only if α-synuclein linked to one fragment is transferred from its parent cell to a recipient cell containing α-synuclein conjugated to the other fragment, Venus fluorescence is observed. Using this system, they detected that cells expressing p62 (C331A) secreted C-terminal hemi-Venus-α-synuclein to the extracellular space leading to uptake in recipient cells expressing an N-terminal hemi-Venus-α-synuclein conjugated protein, suggesting that increased EV-mediated α-synuclein secretion facilitated cell-to-cell spread. Although this mechanism has been supported by multiple previous reports demonstrating that pathological α-synuclein is transported preferentially *via* EVs and that EV-associated α-synuclein is taken up by recipient cells, which in turn may increase α-synuclein induced toxicity in the recipient cells (Emmanouilidou et al., 2010; Danzer et al., 2012; Shi et al., 2014; Fussi et al., 2018; Minakaki et al., 2018; Sepulveda et al., 2022), Oh et al. did not show direct evidence for EV-associated α-synuclein uptake in recipient cells and subsequent seeding. Thus, the relationship between the observed increased secretion of EVs containing α-synuclein and increased cell-to-cell transfer needs to be further explored. Visualization of EV uptake in recipient cells, e.g., through staining of EV membranes using a specific fluorescent dye, such as PKH67 (Kim et al., 2006; Fitzner et al., 2011), or by immunocytochemistry after fixing the cells using EV-specific markers, could provide direct evidence correlating the increased secretion of EV-associated α-synuclein, and uptake followed by seeding in recipient cells.

Previous studies have shown that increased oxidative stress and autophagy dysfunction lead to increased secretion of α-synuclein, possibly in EVs (Danzer et al., 2012) as a compensatory mechanism to the inhibition of autophagic flux. EVs containing α-synuclein have been shown to transport from the brain to the periphery, facilitating their use as a source of biomarkers using a minimally invasive blood draw (Shi et al., 2014; Hornung et al., 2020). As S-nitrosylation of p62 leads to a 3.3-fold increase in EV-mediated α-synuclein secretion, one might expect the concentration of α-synuclein in central nervous system (CNS)-originating blood EVs to be higher in patients with synucleinopathies than in controls. Indeed, recent studies have confirmed that this is the case in patients with PD and multiple system atrophy (MSA) (Shi et al., 2014; Dutta et al., 2021; Jiang et al., 2021) as opposed to direct measurement of α-synuclein in the blood, in which little differences were found between patients and healthy controls. The latter studies showed that α-synuclein concentrations in CNS-originating EVs could distinguish between PD and MSA (Dutta et al., 2021; Jiang et al., 2021), yet whether this is due to different levels of SNO-p62 in these diseases or to other factors is not yet known.

Oxidative stress occurs in a whole organ (or a whole organism) and is expected to affect the whole brain. However, neurodegenerative diseases often affect certain brain areas and/or particular cell types, e.g., the nigrostriatal tract in PD, oligodendrocytes in MSA. In view of the findings of Oh et al., we are curious to whether a correlation can be found between levels of p62 S-nitrosylation and the regions or cell types that are most vulnerable in each disease. For example, early-stage PD is associated with Lewy-body pathology primarily in the brainstem, gradually spreading to the midbrain and affecting dopaminergic neurons in the *substantia nigra pars compacta* (Braak et al., 2003) whereas in LBD, Lewy body accumulation and neurodegeneration are more prominent in cortical structures (Kalaitzakis et al., 2009). Does S-nitrosylation of p62 occur differentially in these brain regions in PD and LBD?

Similar to the deposition of α-synuclein primarily as glial cytoplasmic inclusions in the oligodendrocytes of patients with MSA (Lee et al., 2019), in parkinsonian tauopathies, such as supranuclear palsy (PSP) and corticobasal degeneration (CBD), aggregated, hyperphosphorylated tau inclusions are found not only in in neurons, but also prominently in astrocytes and oligodendrocytes (Ferrer et al., 2014). Oxidative stress generating SNO-p62 would be expected to impair clearance of tau aggregates, similar to the effect in synucleinopathies, and possibly also lead to increased release of pathological forms of tau in EV-dependent and/or independent mechanisms. Are levels of SNO-p62 in the glial cells in these diseases increased compared to those that affect primarily neurons?

In summary, Oh et al. found that nitrosylation of p62 leads to inhibition of autophagy, which in turn increases the extracellular release of α-synuclein, helping to elucidate a new component of the mechanism underlying synucleinopathies and potentially other neurodegenerative diseases. In support of the proposed mechanism, they report higher levels of SNO-p62 in post-mortem human brains, mouse models of synucleinopathy, and human iPSC-derived neurons compared to the appropriate controls. SNO-p62 and its surprising phenotypical analog p62 (C331A) were found to bind LC3 with higher affinity than the unmodified protein. The study's results raise several interesting questions, such as how SNO-p62 affects different brain regions, different cell types and different amyloidogenic proteins in PD, parkinsonian disorders, and more generally, other proteinopathies. It is also interesting to explore what makes p62 (C331A) behave similarly to SNO-p62 despite the distinct steric and hydropathic nature of the side chain at position 331 of these protein isoforms. It is hoped that the study will inspire follow-up investigations that will address these questions and take us a step closer to understanding the processes that cause and propagate neurodegenerative diseases.

## Author contributions

HT and GB: literature search, conception, writing of manuscript, and figure conception. BK, HT, and GB: figure design and illustration. All authors contributed to the article and approved the submitted version.

## Funding

GB acknowledges generous support from Cure Sanfilippo Foundation grant 20215318, the Karen Toffler Charitable Trust, and the Binder Foundation.

## Conflict of interest

The authors declare that the research was conducted in the absence of any commercial or financial relationships that could be construed as a potential conflict of interest.

## Publisher's note

All claims expressed in this article are solely those of the authors and do not necessarily represent those of their affiliated organizations, or those of the publisher, the editors and the reviewers. Any product that may be evaluated in this article, or claim that may be made by its manufacturer, is not guaranteed or endorsed by the publisher.
